# Osmotic stress in colony and planktonic cells of *Pseudomonas putida* mt-2 revealed significant differences in adaptive response mechanisms

**DOI:** 10.1186/s13568-017-0371-8

**Published:** 2017-03-15

**Authors:** Nancy Hachicho, Astrid Birnbaum, Hermann J. Heipieper

**Affiliations:** Department of Environmental Biotechnology, Helmholtz Centre for Environmental Research-UFZ, Permoserstr. 15, 04318 Leipzig, Germany

**Keywords:** *Pseudomonas putida*, Osmotic stress, Agar grown cells, Planktonic cells, Cell surface hydrophobicity, Membrane fatty acids, Stress response

## Abstract

Planktonic cells and those grown on surfaces (or as colony biofilm) are known to show significant differences regarding growth behavior, cell physiology, gene expression and stress tolerance. In order to compare stress behavior of different growth forms, shake cultures for planktonic growth and agar plate cultivation for colony growth, were carried out with the well investigated model organism, *Pseudomonas putida* mt-2. Cells were exposed to sodium chloride to cause osmotic stress as one main environmental stressor bacteria have to cope with when growing in soil. Planktonic cells were more tolerant with a complete inhibition of growth at 0.7 M NaCl, compared to 0.5 M for agar-grown cells. Cell surface hydrophobicity, measured as water contact angles, was significantly higher for agar-grown cells (92°) than for planktonic cells (40°), and increased in the presence of NaCl. Agar-grown cells also showed a significantly higher degree of saturation of membrane fatty acids that increased in the presence of NaCl. These results demonstrate that planktonic and colony grown bacteria show different responses when confronted with osmotic stress suggesting that the tolerance and adaptive mechanisms are dependent on the environmental conditions as well as the initial physiological state.

## Introduction

Planktonic cells and those grown in form of microcolonies or biofilms show significant differences regarding growth behavior, cell physiology, gene expression and stress tolerance. The description of a bacterial strain and its properties are strictly linked to the culture conditions (Vartoukian et al. 2010; de Sarrau et al. 2012). Physiological studies and genome analyses highlight the impressive potential of stress adaptation and morphological or phenotypic variety of bacteria. Phenotypic variations of bacteria lead to enormous differences in cell physiology and biochemistry as well as in tolerance towards environmental stresses (Beveridge et al. 1997; Drenkard and Ausubel 2002; Heipieper et al. 1991; Serra et al. 2013). Many bacteria are capable to grow in form of flagella driven planktonic cells as well as in form of sessile, biofilm creating cells; whereby the way of living is strongly dependent on the environmental conditions (Young et al. 1999; Shrout et al. 2006). However, regular stress tolerance studies with bacteria test the adaptive potential under ideal planktonic growth conditions (Unell et al. 2007; de Carvalho et al. 2014). But except for some very specific studies on pathogenic bacteria (Garcia et al. 2013) our knowledge of bacterial adaptation to environmental stressors when cultivated in different growth conditions is still only partial.

Therefore, the aim of this study was to compare the effect of osmotic stress caused by NaCl on growth and adaptive responses of *Pseudomonas putida* mt-2 cultivated both as colonies grown on agar plates or freely suspended planktonic cells. Next to growth tolerance, changes in cell surface hydrophobicity and membrane fatty acid composition were taken as important adaptive mechanisms to environmental stressors.

## Materials and methods

All experiments were carried out with the bacterial strain *Pseudomonas putida* mt-2 (DSM 6125).

### Liquid cultures

For pre-cultures bacterial colonies were transferred to 50 ml mineral salt media (Hartmans et al. 1989) in 250 ml glass flasks with screw caps with 4 g l^−1^ of Na_2_-succinate, and 1 g l^−1^ of yeast extract as carbon and energy source, oxygen was always sufficiently present under these conditions. All liquid cultures were incubated at 30 °C and 160 rpm in an orbital shaker. Pre-cultures were incubated overnight and thereafter used to inoculate samples with 5 ml sterile mineral salt media supplemented with sodium chloride to different concentrations (0, 0.1, 0.3, 0.5, 0.7 and 1.0 M) in 20 ml glass vials with screw caps. Cell growth was periodically monitored by optical cell density measurement at 560 nm (OD_560_) using a Perkin Elmer UV/VIS Spectrophotometer (UV–Vis Spectrometer: Lambda2S, Perkin/Elmer, Waltham, USA). The initial optical density was set to 0.05. Cells were harvested in the late log phase by centrifugation at 11,000×*g* for 15 min and washed twice with 2 ml KNO_3_ (10 mM, pH 7.0). The washed biomass was split and one half was re-suspended in 2 ml KNO_3_ and stored at 5 °C for 18 h before contact angle measurement. The second half was concentrated by centrifugation and the biomass pellet was stored at −20 °C until fatty acid extraction was performed.

### Agar plates

For growth inhibition experiments on agar plates the same mineral salt medium as for liquid cultures was used with supplementation with 2% agar to obtain similar conditions. After sterilization, 5 ml portions sterile mineral salt agar supplemented with sodium chloride to different concentrations were transferred to the wells of a six-well-plate. The solidified agar was inoculated with a drop of the pre-culture (3 µl) in the centre of each agar plate and incubated at 30 °C for 8, 24 and 32 h. The whole colonies/biofilms developed were washed down quantitatively from the agar surfaces with 2 ml KNO_3_ (10 mM, pH 7.0). For cell harvesting the 2 ml KNO_3_ were gently pipetted on the agar surface into the well of the six-well-plate and resumed multiple times until the colonies were washed down from the agar surface and re-suspended in the 2 ml KNO_3_. The cell suspension was used for optical density measurement as described above (influence of agar on the optical density is negligible which was observed in preliminary investigations, data not shown) and the same samples were used for contact angle analysis and fatty acid extraction after washed twice as described above. The washed biomass was split and one half was re-suspended in 2 ml KNO_3_ and stored at 5 °C for 18 h before contact angle measurement. The second half was concentrated by centrifugation and the biomass pellet was stored at −20 °C until fatty acid extraction was performed.

### Water activity measurement

Measurement of water activity a_w_ of sterile liquid and solid samples was performed with the LabMaster^®^-aw instrument (Novasina AG, Switzerland). The measurement is based on resistive electrolytic humidity analysis. System parameters were set at 5 min stabile observation time for temperature and water activity and measurement was performed at 25 °C. A volume of 5 ml per sample was filled in a dry and clean sample cup (Ø 40 mm, 12 mm, polypropylene, ePW sample cups, Novasina) and each sample was treated 10 min in the pre-condition chamber of the instrument before measurement.

### Growth inhibition

Growth inhibition was measured by calculating the growth rate µ [h^−1^] of each culture in the growth phase as previously described (Keweloh et al. 1989). Growth rates were expressed as relative growth to control cultures and plotted against the stressor concentration. The minimal inhibitory concentration (MIC) was defined as the concentration of the stressor that completely blocked bacterial growth according to Keweloh et al. (1989).

### Contact angle measurement

Washed biomass was suspended in 20 ml KNO_3_ and the suspension was filtered through a membrane filter (0.45 µm, Ø 25 mm, cellulose nitrate, NC 45, Whatman) to produce a bacterial-covered filter surface. The wet filters were applied to divided microscope slides prepared with double-sided adhesive tape to fix the filters and dried for 2 h. Measurement was performed using the drop shape analysis system (DSA 100, Krüss GmbH, Germany) by video documentation of the fall down of a water drop (3 µl volume, 40 µl s^−1^ rate) on the bacterial-covered filter surface The contact angle θ [°] between water/bacterial surface/air was measured by image analysis with the DSA software using the circle fitting mode. The procedure was performed four times per filter and the average was calculated.

### Lipid extraction and FAME synthesis

The lipids were extracted with chloroform/methanol/water as described by Bligh and Dyer (1959). Fatty acid methyl esters (FAME) were prepared by incubation for 15 min at 95 °C in boron trifluoride/methanol applying the method of Morrison and Smith (1964). FAMEs were finally extracted with hexane.

### Analysis of membrane fatty acid pattern by GC-FID

Analysis of FAME was performed using a quadruple GC System (Agilent Technologies, 6890N Network GC System, 7683 Series Injector) equipped with a split/splitless injector. A CP-Sil 88 capillary column (Chrompack, Middelburg, The Netherlands; length, 50 m; inner diameter, 0.25 mm; 0.25 mm film) was used for the separation of the FAME. The peak areas of the fatty acids were used to determine their relative amounts. The peaks were compared to commercial standards (Supelco, Bellefonte) for qualitative analysis. The relative amounts were used to calculate the *degree of saturation* of membrane fatty acids. Hereby, the cyclopropane fatty acid 17*cyclo* which was present in significantly different amounts in both types of cultivation was taken as unsaturated fatty acid because of their quite similar transition temperature (Cronan 2002).

1 $$ Degree\;of\;Saturation = \frac{C16{:}0 + C18{:}0}{C16{:}1 + C17{:}0cyclo + C18{:}1} $$

### Statistics

All experiments were carried out four times. All figures show mean values with the corresponding standard deviations as error bars.

## Results

**Fig. 1:**
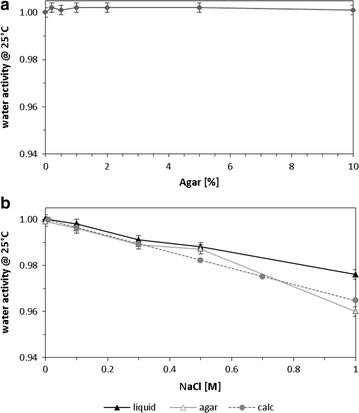
Effect of agar (**a**) and sodium chloride (**b**) on the water activity. *Filled triangles* liquid medium, *open triangles* agar plates (2% agar), and *filled diamonds* calculated values

The present study consisted of two batch cultivation techniques performed on agar plates and in liquid cultures under similar conditions. In order to guarantee the same osmotic strength on agar plates and in liquid cultures, the effect of both agar–agar and sodium chloride on the water activity a_w_ was measured to prevent unknown, cumulative stress effects. Water activity was stable over an agar concentration range from 0 to 10%. For sodium chloride, a linear correlation between salt concentration and decrease in water activity was found with similar values for liquid cultures and agar plates were a_w_ decreased from 1 to 0.965, proving that the presence of agar had no significant effect on the a_w_ (Fig. 1).

**Fig. 2:**
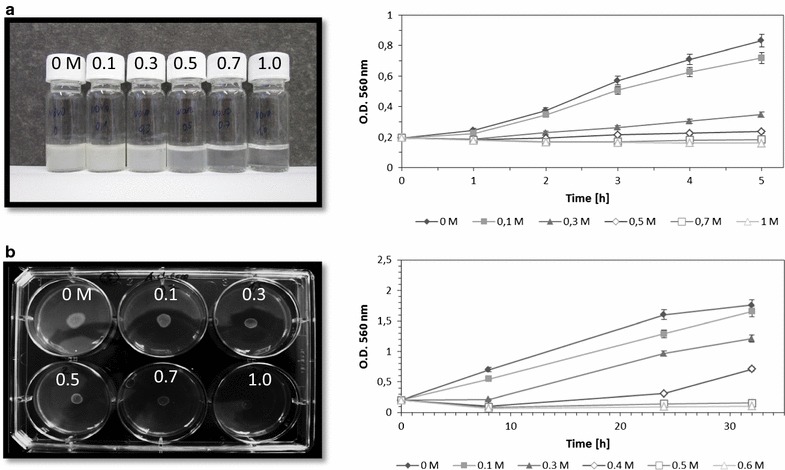
Growth curves of *Pseudomonas putida* mt-2 cultivated in the two different experimental setups. **a** Growth inhibition in liquid cultures with mineral medium and increasing concentrations of sodium chloride. **b** Growth inhibition on 2% agar in 6-well-plates with mineral medium and increasing concentrations of sodium chloride. Photographs of the cultures were taken after 5 h incubation for planktonic and after 32 h for agar-grown cells

The cells of model strain *P. putida* mt-2 were initially exposed to NaCl in different concentrations and harvested in the late growth phase (Fig. 2). The effect of NaCl on growth, surface hydrophobicity and membrane composition was investigated for both growth conditions.

The bacterial growth was monitored by periodical measurement of optical density. Resulting growth curves (Fig. 2) showed a NaCl induced, dose dependent growth inhibition. The growth rate of each culture was calculated and plotted against the sodium chloride concentration (Fig. 4a). The growth rates µ were 0.25 ± 0.015 h^−1^ for liquid cultures without salt supplementation and 0.07 ± 0.003 h^−1^ for colony grown cells. Planktonic cells showed a linear growth inhibition with minimal inhibitory concentration (MIC) at 0.7 M NaCl, whereas the colony forming cells displayed a slightly inhibition effect at low salt concentrations followed by a rapid growth slump (Fig. 4a). The MIC for agar grown cells was 0.5 M. The effective concentration which induces a growth inhibition of 50% (EC_50_) was for both forms of growth at 0.4 M NaCl. In both systems tested, growth inhibition was absolutely reversible as only bacteriostatic concentrations of NaCl were added; the measured optical densities and cell titers, respectively, correlated with the corresponding CFU (data not shown).

The effect of NaCl on the cell surface hydrophobicity was analysed and expressed as water contact angle (Fig. 4b). For planktonic cells, the contact angle increased from 40° to 75° with increasing salt concentrations. Contrary to that, the colony grown cells showed already initial contact angles of 92°. In the presence of salt concentrations up to 0.3 M, the contact angles increased to 107°, followed by strong decrease to 48° with further increasing salt concentrations.

**Fig. 3:**
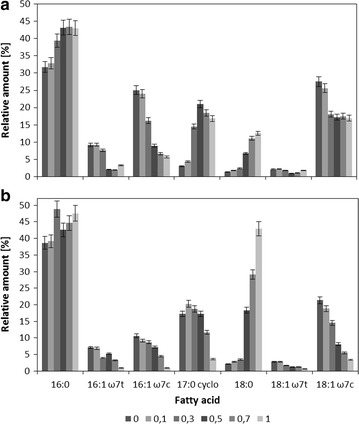
Membrane fatty acid composition of *Pseudomonas putida* mt-2 grown in liquid cultures (**a**) and on agar plates (**b**) with different concentrations of sodium chloride

In addition, the effect of osmotic stress on the composition of the membrane fatty acids was investigated by lipid extraction and analysis of fatty acid methyl esters (FAME). The membrane fatty acid pattern of strain mt-2 consisted of seven dominant fatty acids. The saturated fatty acids C16:0 and C18:0, the mono-unsaturated fatty acids C16:1 ω7*trans*, C16:1 ω7*cis*, C18:1 ω7*trans* and C18:1 ω7*cis* and C17:0*cyclo* were found as the predominant lipid components (Fig. 3). Significant differences in these fatty acid patterns were observed between both growth conditions tested. For planktonic cells C16:0, 16:1*cis*, and C18:1*cis* fatty acids comprised 85% of the lipid component whereas for agar grown cells C16:0 and 18:1 fatty acids comprised 80% of the lipid component. In the presence of NaCl, the amount of C17:0*cyclo* increased from <5 to 20% also the saturated C16:0 and C18:0 increased when cells were grown in liquid cultures. In cells grown on agar plates, the relative amount of saturated C18:0 increased from 3 to 45% while the amount of C17:0*cyclo* decreased from 30 to 10% after growth in the presence of high concentrations of NaCl.

**Fig. 4:**
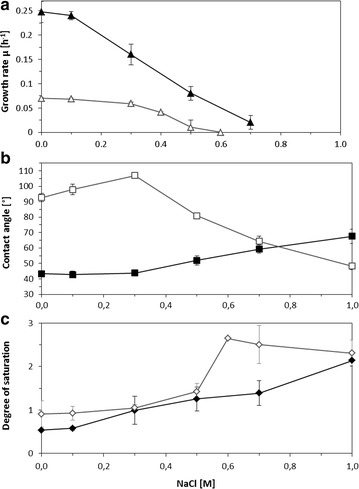
Effect of sodium chloride on growth, cell surface hydrophobicity and membrane fatty acids. Growth inhibition (**a**), contact angle (**b**) and degree of saturation of membrane fatty acids (**c**) of *Pseudomonas putida* mt-2 after growth in liquid cultures (*filled symbols*) and on agar plates (*open symbols*) with different concentrations of sodium chloride

The relative amounts were used to calculate the degree of saturation of membrane fatty acids (Fig. 4c). For both cultivation techniques a dose dependent increase was found. The initial value was 0.5 for cells grown in liquid cultures and 0.9 for those grown in form of colonies. Planktonic cells showed an increase of the degree of saturation up to 2.0 whereas for colony grown cells the degree of saturation increased to 2.8 in presence of high salt concentrations.

## Discussion

With the experimental approach used in our studies we could show that planktonic and colony grown cells of the well-investigated strain *P. putida* mt-2 show different responses when confronted with osmotic stress which suggests that the tolerance is linked to the environmental conditions and the initial physiological state.

Despite about 3.5 times lower growth rates of agar grown cells, a dose depended growth inhibition was observed for both forms of growth with identical EC_50_ values but different MIC values for sodium chloride. NaCl is often used for the simulation of osmotic stress and is also known to reduce the water activity of the medium inducing water stress (Kets et al. 1996; Heipieper et al. 1996; Halverson and Firestone 2000). The initial exposure of the cell to the stressor is in contrast to other stress studies were the stressor is added during the early exponential growth phase (Neumann et al. 2006; Baumgarten et al. 2012). Regarding natural conditions in soil systems, it can be assumed that exponential growth is often inhibited due to unfavorable conditions like nutrient limitations, reduced water availability, temperature and/or competition events. Therefore, the stressor was added prior inoculation with bacterial cells. The cells showed identical tolerance, at least for EC50 values which were 0.4 M NaCl for both tested types of cells despite a threefold higher growth rate when grown in liquid cultures. This is probably based on effective osmoregulatory mechanisms which prevent the cells from dehydration and plasmolysis (Kempf and Bremer 1998), where initial intracellular accumulation of potassium ions followed by synthesis or uptake of compatible solutes takes place (Dinnbier et al. 1988; Kets et al. 1996).

The adaptive response to the osmotic stress was investigated and compared for both culture conditions. Surface properties of bacteria like hydrophobicity are important parameters with respect to biofilm formation, environmental interactions and stress response (Wick et al. 2002; Neumann et al. 2006; Harms et al. 2010). The hydrophobicity of bacteria depends on the molecular composition of the cell surface and is an important factor for the adhesion and formation of biofilm and/or cell aggregates (van Loosdrecht et al. 1987). In general, water contact angles lower 20° represent hydrophilic surfaces, values between 20° and 70° mean intermediate hydrophilic/hydrophobic, whereas hydrophobic surfaces show contact angles >70° (van Loosdrecht et al. 1987). In addition, bacteria are able to modify their surface hydrophobicity in order to react to changing environmental conditions (Wick et al. 2002; de Carvalho et al. 2009). The modification of cell surface hydrophobicity has been often reported in combination with stress studies on bacteria (Neumann et al. 2006; Baumgarten et al. 2012). The results show an intermediary hydrophilic surface for planktonic grown cells, while colony grown cells were highly hydrophobic. Reason for this very high cell surface hydrophobicity of agar-grown cells is the fact that cells in bacterial colonies are attached to each other very closely which is mainly done by increasing their cell surface hydrophobicity (Baumgarten et al. 2012). An increase in cell surface hydrophobicity is known to be key factor for biofilm formation and adhesion to surfaces (van Loosdrecht et al. 1987). A change from hydrophilic to hydrophobic surface properties allows bacteria to survive osmotic attacks by formation of microcolonies which are known to show higher tolerance towards all kinds of environmental stressors (Keweloh et al. 1989; Heipieper et al. 1991; Baumgarten et al. 2012). A possible explanation of the observed decrease in contact angles of agar grown cells in the presence of NaCl might be the fact that cells are also known to be able to leave the community as planktonic cells in a process called dispersal.

The membrane fatty acid composition was also strongly influenced by the cultivation technique indicating again the differences of the physiological state of the cells. The degree of saturation of agar-grown cells was generally higher than that of planktonic cells. Like for the higher cell surface hydrophobicity, this can be explained by the colony or biofilm organization of the bacteria in this growth form. Cells grown in form of biofilms are known for the increased rigidity of their membrane bilayers (de Carvalho et al. 2009). In addition, cyclopropane fatty acids were initially present in high amounts in cells grown on agar plates while planktonic cells increased the relative amounts of C17:0*cyclo* after growth in the presence of high salt concentrations. This can be explained by the regulation of the synthesis of cyclopropane fatty acids from the corresponding *cis*-unsaturated fatty acids which occurs either when cell enter the stationary phase or in the presence of osmotic stressors such as NaCl (Cronan 2002). Thus, this is another indication about the physiological status of agar-grown cell as slow growing cells. The membrane fatty acids in both forms of cultivation, showed an increase in the relative amount of saturated fatty acids and a corresponding decrease in unsaturated fatty acids. Regularly this is expressed as the degree of saturation which increased significantly. These results are in agreement with earlier starvation experiments performed in porous media (Kieft et al. 1994). Higher amounts of saturated fatty acids increase membrane packaging and rigidity. The gaps between the phospholipid molecules became smaller due to the optimized steric arrangement of fatty acid chains. The consequence is a dense and rigid membrane which is probably more leak-proof and able to prevent water loss induced by osmotic stress. The observation that the relative amount of both *cis*- and *trans*-unsaturated fatty acids decreased is in contrast to previously descripted experiments where osmotic shock induces the alteration of *trans* to *cis* isomers (Halverson and Firestone 2000). The *cis*–*trans* isomerase in *P. putida* is known to be an urgent stress adaptive mechanism that mainly works in the absence of other membrane related responses (Heipieper et al. 2003).

The competition between bacteria in an environment can be triggered by abiotic factors such as temperature, pH, and osmotic stress (Thomas and Wimpenny 1996). In general, biofilms or microcolonies as they occur in agar-grown cells, show advantages over planktonic cells regarding tolerance of environmental stress such as an increased tolerance to antibiotics, solvents and physical stresses. Cells present in such structures are also known to be able to leave the community as planktonic cells in a process called dispersal, this allows to occupy other niches (Beveridge et al. 1997; Hall-Stoodley et al. 2004). In addition, physiological differentiation and gene expression of single cells within a bacterial microcolony was described in a current study which underlines the potential of physiological adaptation within one bacterial strain (Serra et al. 2013).

The different outcomes of similar experiments underline once more the challenge of comparability which is based on differences in culture conditions, growth phase, harvesting and extraction protocol which may all influence the physiological state of bacterial cells. The results indicate adaptive response mechanism on the level of membrane composition and cell surface hydrophobicity for colony and planktonic grown cells of *P. putida* mt-2 but with different trends in dependence of environmental and initial physiological conditions. The experimental approach used in the present work allows comparing the different outcomes of similar physiological studies and outlining the capacity of adaptive stress response reactions of single species.

## Abbreviations

CFU: colony forming units
EC50: effective concentration 50%
FAME: fatty acid methyl esters
FID: flame ionisation detector
MIC: minimal inhibitory concentration
NaCl: sodium chloride
PLFA: phospholipid fatty acids
